# Natural antibody responses to the capsid protein in sera of Dengue infected patients from Sri Lanka

**DOI:** 10.1371/journal.pone.0178009

**Published:** 2017-06-05

**Authors:** Mahesha N. Nadugala, Chandima Jeewandara, Gathsaurie N. Malavige, Prasad H. Premaratne, Charitha L. Goonasekara

**Affiliations:** 1 Faculty of Medicine, General Sir John Kotelawala Defence University, Ratmalana, Sri Lanka; 2 Centre for Dengue Research, University of Sri Jayawardanapura, Gangodawila, Sri Lanka; Central University of Tamil Nadu, INDIA

## Abstract

This study aims to characterize the antigenicity of the Capsid (C) protein and the human antibody responses to C protein from the four dengue virus (DENV) serotypes. Parker hydrophilicity prediction, Emini surface accessibility prediction and Karplus & Schulz flexibility predictions were used to bioinformatically characterize antigenicity. The human antibody response to C protein was assessed by ELISA using immune sera and an array of overlapping DENV2 C peptides. DENV2 C protein peptides P1 (located on C protein at 2–18 a.a), P11 (79–95 a.a) and P12 (86–101 a.a) were recognized by most individuals exposed to infections with only one of the 4 DENV serotypes as well as people exposed to infections with two serotypes. These conserved peptide epitopes are located on the amino (1–40 a.a) and carboxy (70–100 a.a) terminal regions of C protein, which were predicted to be antigenic using different bioinformatic tools. DENV2 C peptide P6 (39–56 a.a) was recognized by all individuals exposed to DENV2 infections, some individuals exposed to DENV4 infections and none of the individuals exposed to DENV1 or 3 infections. Thus, unlike C peptides P1, P11 and P12, which contain epitopes, recognized by DENV serotype cross-reactive antibodies, DENV2 peptide P6 contains an epitope that is preferentially recognized by antibodies in people exposed to this serotype compared to other serotypes. We discuss our results in the context of the known structure of C protein and recent work on the human B-cell response to DENV infection.

## Introduction

Dengue viruses (DENV), arthropod-borne viruses, cause a significant global health burden. A recent study estimated around 390 million dengue infections annually, resulting in approximately 100 million symptomatic cases and around 25,000 deaths [1]. During the last 60 years the incidence of dengue cases reported to WHO has increased 30-fold, with a much increased geographic range and expansion from urban to rural settings. Although the number of cases increases annually, there is only one partially efficacious vaccine registered for prevention [2], and many basic aspects of the viral biology remain elusive.

The genome of DENV is a single, positive-stranded RNA that encodes three structural proteins: envelope (E), premembrane (prM) and capsid (C) and seven nonstructural proteins (NS) [3]. The four distinct serotypes (DENV1 to 4) show 67–75% sequence homology [4]. Infection with dengue virus causes symptoms ranging from acute febrile illness to severe manifestations like hemoerragues and organ failures.

C protein of DENV is a small, highly basic 12-kDa protein required for virion assembly. The C protein is involved in the proper encapsidation of the RNA genome, resulting in a spherical nucleocapsid with a single copy of the ssRNA molecule [5]. The DENV C protein is composed of four alpha helices with an unstructured amino terminus and forms antiparallel homodimers. In this configuration, one face of the capsid dimer is highly charged, whereas the opposite face contains a hydrophobic cleft [4]. C protein forms homodimers in solution and oligomerizes upon nucleic acid binding. Viral particle reconstructions have suggested that the capsid works as a nucleoprotein that condenses and covers the viral RNA [5, 6, 7]. In the virion, it is also known that the viral nucleocapsid is not exposed on its surface [3, 5]; therefore the C proteins are less likely to stimulate B-cells during viral infection. However, antibodies to C protein have been detected in sera of dengue infected patients in several studies [8, 9, 10]. Most of the studies have been focused only on the antibody responses of one DENV serotype [8, 9, 10]. Here we define DENV serotype cross- reactive and type-specific epitopes on C protein using immune sera from people exposed to different DENV serotypes.

The present study evaluates the entire C protein sequences of all four DENV serotypes for its antigenicity, by means of *in-silico* tools. The study further evaluates the immunogenicity of DENV2 capsid peptides by measuring its natural antibody responses of DENV infected patients from all four serotypes. This paper, therefore, describes the characterization of the C protein of DENV based on bioinformatical prediction of the antigenicity of the C protein and the natural antibody responses against the C protein of the individuals who have been previously infected with all four DENV serotypes. Our results reinforce the usefulness of *in-silico* tools in protein epitopes prediction and further provide useful evidences for the potential use of C protein epitopes in dengue virus diagnostics and vaccine development.

## Materials and methods

### Bioinformatic analysis of C protein

#### Retrieving the protein sequences

The C protein sequences from 200 variants (50 each from DENV1, DENV2, DENV3 and DENV4) were retrieved from National Center for Biotechnology information (NCBI) (http://www.ncbi.nlm.nih.gov/), while excluding isolates with partial sequences as described in Nadugala et al. [11]. The retrieved data set represents a wide geographical coverage including countries from South Asia, East Asia, America and Africa and a time span of approximately 50 years (isolates from 1963–2014). The variable and conserved regions were compared among the downloaded isolates after aligning the isolates using Clustal W on MEGA6 (http://www.megasoftware.net/).

#### Conservation analysis and phylogenetic analysis

Detailed conservation analysis of the whole C protein was carried out by Conservation Analysis tool available in IEDB resource (http://www.iedb.org) as described by Nadugala et al. [11]. Conservancy was measured at three different levels: (i) within a particular serotype (intra-serotype conservancy) (ii) between two pairs of serotypes and (iii) among the all four serotypes (pan-serotype conservancy). A phylogenetic tree of the entire C protein sequences of isolates was constructed using neighbor-joining method on MEGA6, to elucidate the evolutionary distance of isolates based on the variability in the C protein sequences.

#### Antigenicity prediction of the entire C protein

The C protein sequences of four serotypes were characterized for their antigenic properties using three Bioinformatic tools; namely Parker hydrophilicity prediction [12], Emini surface accessibility prediction [13] and Karplus & Schulz flexibility prediction [14] in IEDB resources. Parameters such as hydrophilicity, flexibility, accessibility, turns, exposed surface, polarity and antigenic propensity of polypeptides chains have been correlated with the location of antigenic regions. This has led to a search for empirical rules that would allow the position of antigenic regions from certain features of the protein sequence. All prediction calculations are based on propensity scales for each of the 20 amino acids. Each scale consists of 20 values assigned to each of the amino acid residues on the basis of their relative propensity to possess the property described by the scale. A similar widow size of 7 a.a. and a threshold level of1 were maintained in all prediction methods. Individual C protein sequences were uploaded separately to each tool in order to obtain the profile for each sequence. Additionally, the prediction of conformational epitopes of the C protein was carried out using Ellipro tool (derived from Ellipsoid and Protrusion) (http://tools.iedb.org/ellipro), as described by Nadugala et al. [11]

### Enzyme linked immune sorbent assay (ELISA)

#### Collection of sera

DENV immune sera were obtained from sixty seropositive healthy volunteers residing in Sri Lanka who had experienced natural DENV infections. Infection with dengue was confirmed by NS1, IgM/ IgG ELISA. The serotype of the previous dengue infections of these subjects have been determined as previously described by using cultured T-cell ELISpot assays, using a panel of peptides which were found to be highly conserved within a serotype and unique to that serotype, therefore, specifically detects an infection by a given dengue virus serotype [15,16]. Individuals whose T-cells responded to peptides that were unique to a particular serotype were considered as being infected with only that particular serotype. Those who responded to two serotypes were considered as being previously infected with those two serotypes. Out of the sixty samples selected for the study, 48 were from healthy volunteers who had only responded to peptides of one dengue viral serotype and therefore considered to have been previously exposed to infections with only one of the 4 DENV serotypes (n = 12 per serotype). The remaining 12 samples were from healthy volunteers who responded to peptides of 2 dengue viral serotypes and were considered to have been previously infected with two DENV serotypes. In addition, sera from twelve dengue naïve volunteers were also obtained to use as controls. Inclusion criteria for selecting volunteers are as follows; healthy people between 10–70 years of age, carrying antibodies against one or two serotypes of DENV. Debilitated/bed ridden peoples and peoples below 10 and over 70 years of age were excluded in this study.

The approval for protocols on recruiting volunteers and collection of blood samples was obtained by the Institutional Ethical Review Boards of University of Sri Jayawardanepura and General Sir John Kotelawela Defence Universities (RP/2014/01). In addition, informed written consent was obtained from subjects prior to the collection of blood. Pooled sera consisting of ten samples representing all four serotypes were used to prepare the positive control serum sample and ten naive samples were used to prepare negative control sample.

#### Capsid peptide array

In order to carry out a comprehensive analysis of the antibody responses of the whole capsid, we selected a peptide array that covers the entire C protein sequence for ELISA assays. A 14-peptides array spanning the entire C protein of DENV2 (strain New Guinea C) was used. Peptides were of 15 to 18 a.a. in length, with 10 a.a. overlaps. A mixture of peptides, covering the entire DENV 4 E protein (Strain Singapore/8976/1195 (NR-9229) was used as a control. The two (C and E protein) peptides arrays were obtained through the NIH Biodefense and Emerging Infections Research Repository, NIAID, NIH.

#### Indirect ELISA assay

For ELISA [17], 96-well polystyrene plates (Sigma, USA) were used. Peptides were added at 1.0μg/well/100μl in a carbonate–biocarbonate buffer (pH 9.6) in duplicate and incubate at 4^°^C for 12h. After the peptide solution was flicked off from wells, the plate was blocked with 200 μl of blocking buffer (10% skimmed milk powder in PBS, pH 7.4, with 0.05% Tween-PBST) at 37^°^C for 2h. Subsequently the plate was washed three times with PBST. Serum samples were diluted 1/100 in blocking buffer, and 100μl of diluted serum was added to each well. The plate was incubated at 37^°^C for 1h and washed three times with PBST. Anti-Human IgG (whole molecule)-Peroxidase antibody produced in rabbit (Sigma, USA) was diluted 1/1000 blocking solution and added to wells. Then the wells were incubated at 37^°^C for 1h, followed by three washes with PBST. 100μl of the substrate solution was then added (10ml of 0.1 citric acid buffer (pH 5.0), 250μl of ABST stock solution (100mg of ABTS in 4.5ml deionised water) and 50μl of H_2_O_2_. Samples were then incubated at room temperature for 20min. The plates were read with a microplate reader with a 450nm filter. The final absorbance result values for each serum sample correspond to the arithmetic mean of the duplicate measurements.The cut-off value for positive immune response was defined using following formula: Cut-off = Mean OD of negative samples + 3 standard deviation (S.D).

#### Statistical analyses

The mean OD values obtained for each combination of serotype and the peptide were subjected to General Linear Models (GLM) and Least square(LS) mean separation procedure in SAS 9.1 (SAS Institute, Cary, NC, USA).

## Results

### Bioinformatic analysis of C protein

#### Conservation analysis of the entire C protein

**Fig 1:**
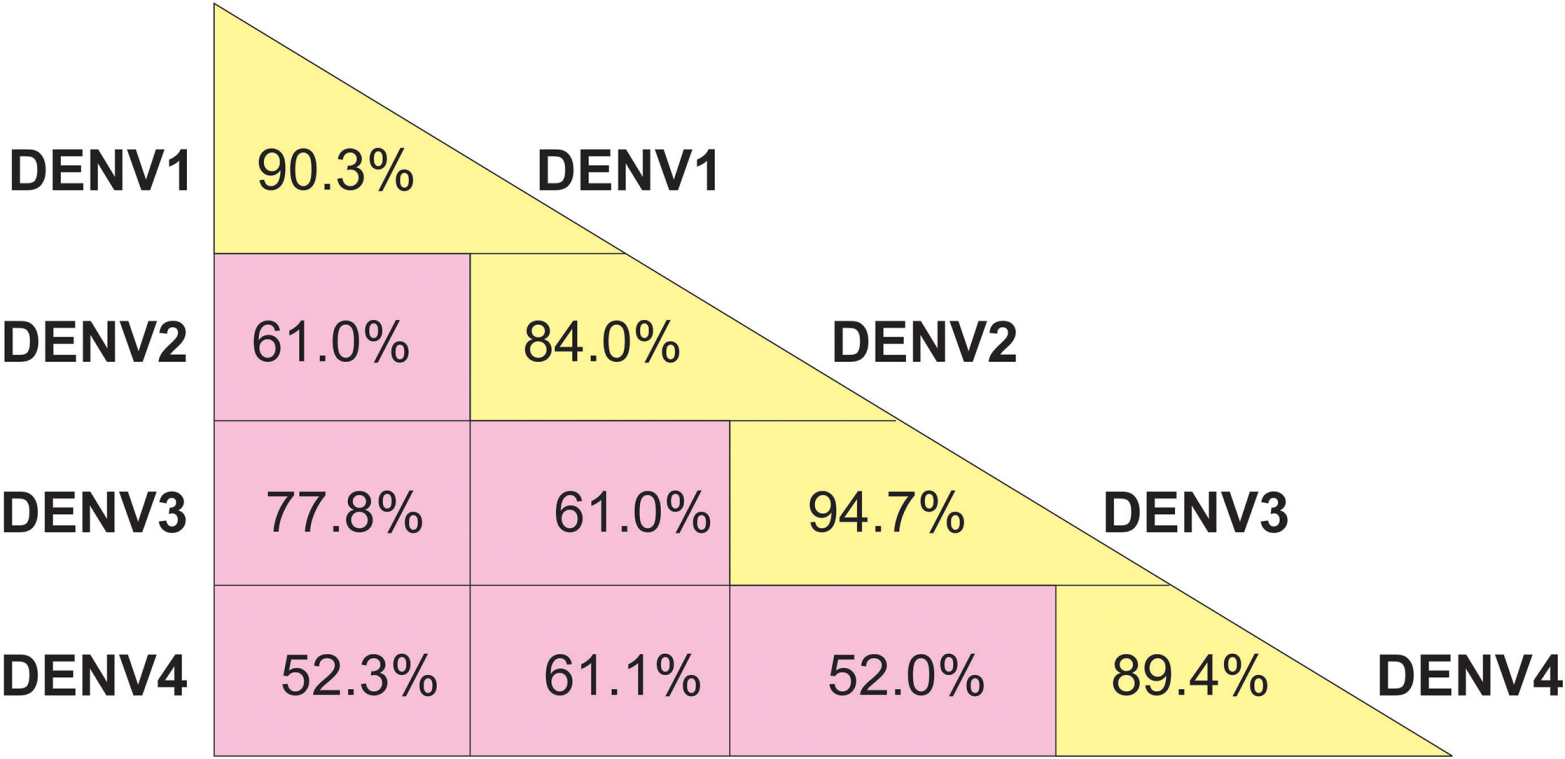
Conservation* analysis of entire C protein. *The conservations ranged from a minimum percentage up to a maximum of 100%.Tabulated percentages are the minimum percentage of conservations.

Conservation analysis on the entire C protein sequences of different DENV isolates is displayed in Fig 1. The level of amino acid similarity among all four serotypes (pan–serotype conservancy) of the entire C protein is 52%. However, a high percentage of intra-serotype conservation was observed within each of the four DENV serotypes (diagonal axis of Fig 1). The percentage values were above 84% in all serotypes. The highest level of percentage intra-serotype conservancy of 94.7% was observed for DENV3, which indicates a higher level of genetic similarity among the isolates of DENV3 serotype used for the study. The lowest percentage of intra-serotypes conservancy (84%) was shown by DENV2. The level of conservation between different pairs of DENV serotypes was also measured, which is also shown in Fig 1. Accordingly, the highest sequence similarity was observed between DENV1 and DENV3.

#### Phylogenetic analysis of the entire C protein

**Fig 2:**
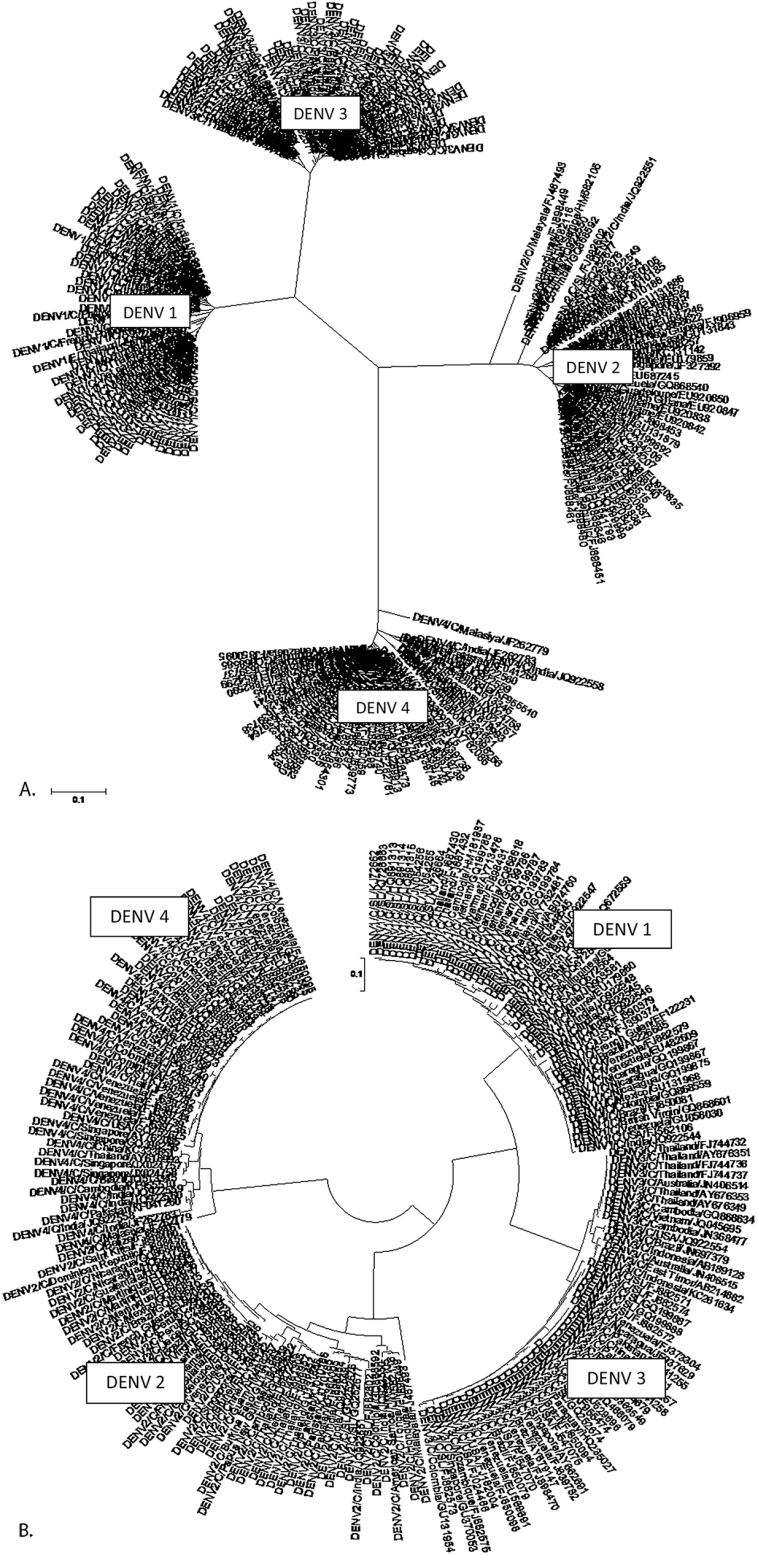
Phylogenetic tree of the entire C protein sequences of four DENV serotypes; A. branch type-radiation B. branch type-circular

The Phylogenetic tree constructed for the same DENV sequences clearly shows the genetic relationship of those isolates within and across the serotypes (Fig 2). Isolates from each serotype cluster into four distance separated groups (Fig 2A). This type of arrangement agrees with the observed low level of pan-serotype conservation and higher levels of intra-serotype conservations of the dengue C protein. DENV3 isolates appears to have organized into the least number of subgroups within the serotype, demonstrating its high intra-serotype conservation (Fig 2B). Further, the two serotypes, DENV1 and DENV3, branches off from same origin and groups closely to each other than to the other two serotypes, indicating their phylogenetic closeness.

#### Antigenicity prediction of the entire C protein

**Fig 3:**
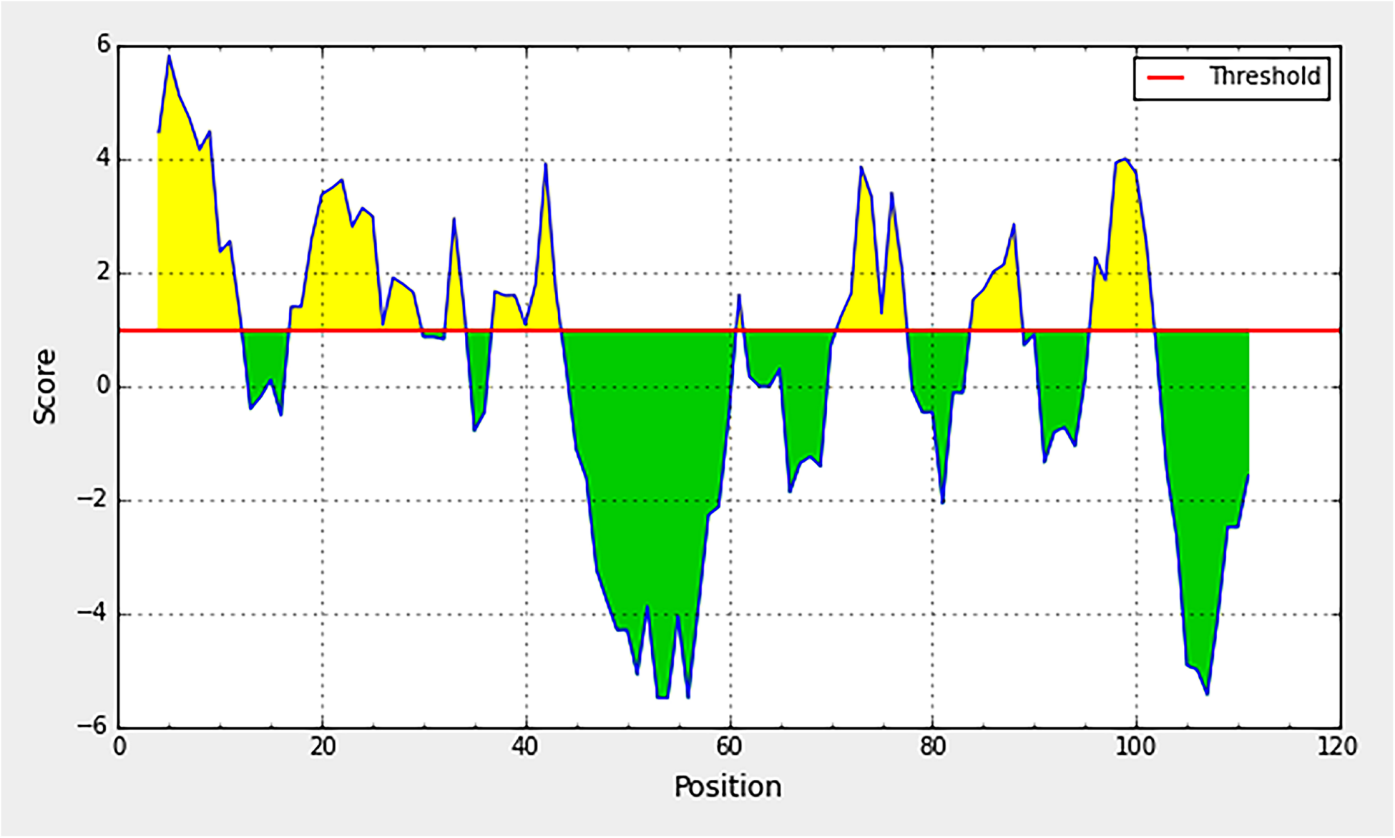
Parker hydrophilicity profile of the C protein. The horizontal axis indicates amino acids residue number and the vertical axis indicates the hydropathy. The profile shown is the output of the sequence of DENV1 isolate AY713476. This profile was 90% similar across all 200 isolates used for the analysis.

**Fig 4:**
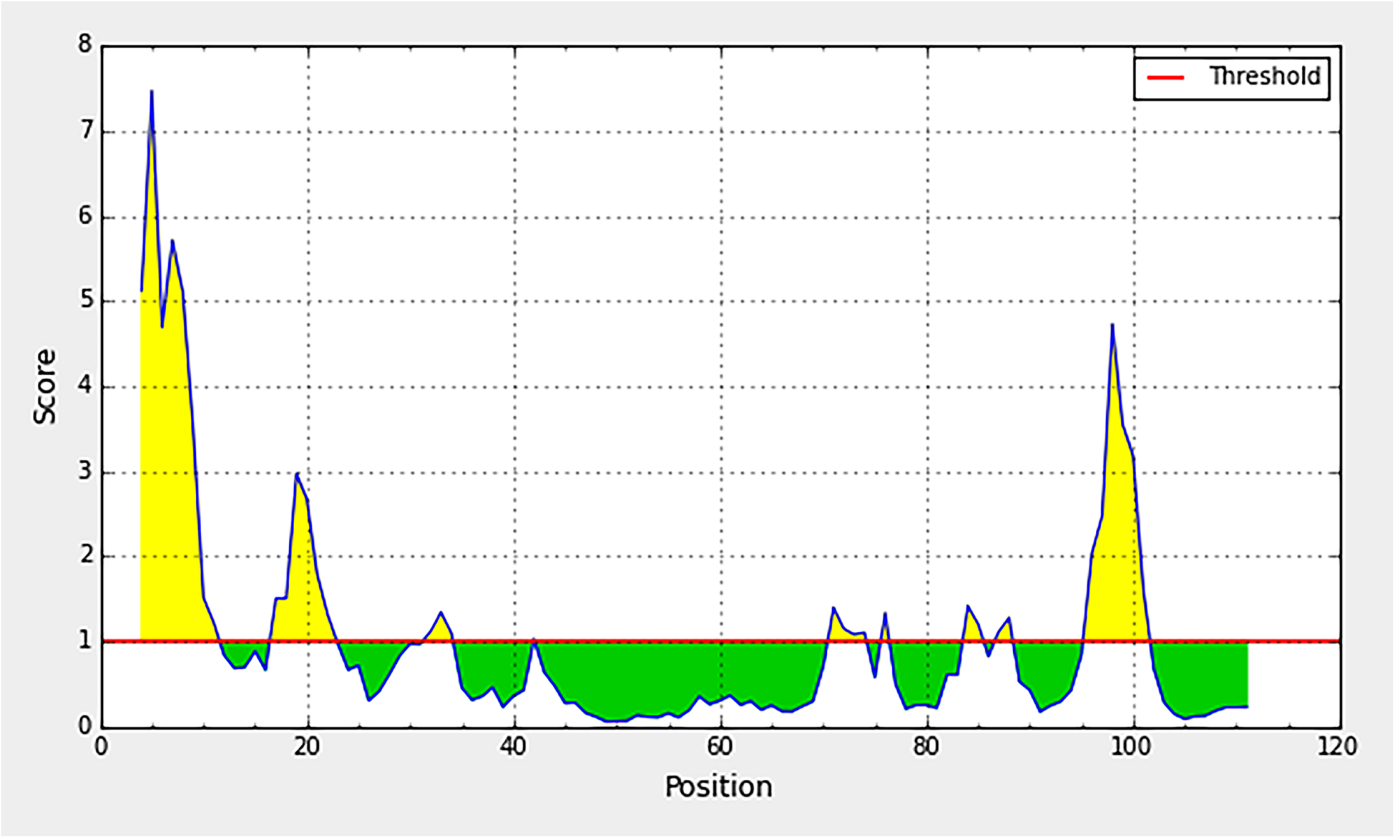
Emini surface accessibility profile of the C protein. The horizontal axis indicates amino acids residue number and the vertical axis indicates the surface accessibility scores. The profile shown is the output of the sequence of DENV1 isolate AY713476. This profile was 90% similar across all 200 isolates used for the analysis.

**Fig 5:**
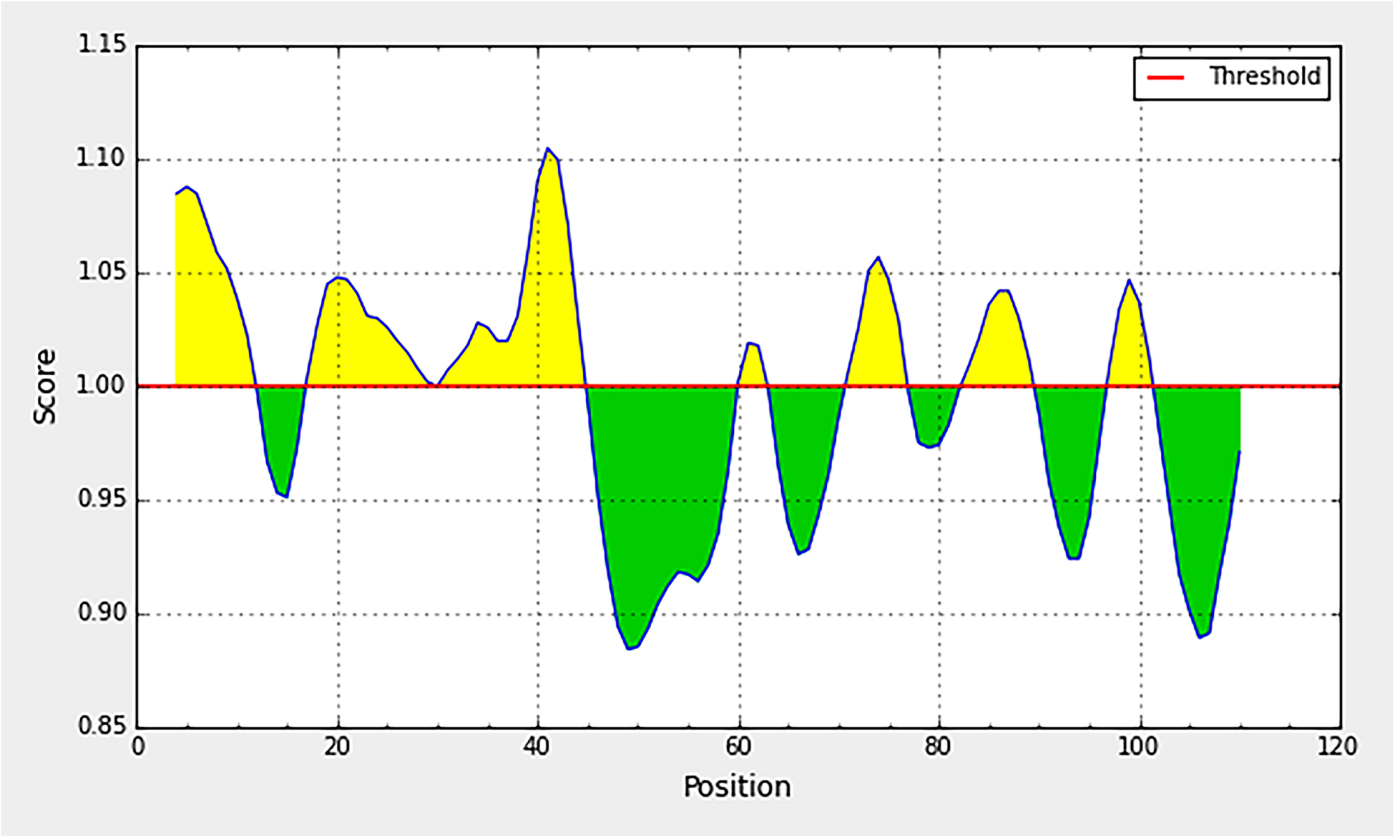
Karplus & Schulz flexibility profile of the C protein. The horizontal axis indicates amino acids residue number and the vertical axis indicates the flexibility scores. The profile shown is the output of the sequence of DENV1 isolate AY713476. This profile was 90% similar across all 200 isolates used for the analysis.

The C protein sequences of the 200 DENVs retrieved from the NCBI were bioinformatically characterized to identify regions predicted to be antigenic. Hydrophilicity, surface accessibility and flexibility of the amino acid residues, were used for evaluating the antigenic regions of the entire C protein, as visualized in the profiles given in Figs 3, 4 and 5. All the profiles are computer stimulated outputs based on the uploaded DENV C protein sequence. A given profile was 90% similar with each other, across all 200 isolates used for the analysis. The profiles given in the figures were outputs of the sequence of DENV2 isolate AAA42941.

Hydrophilicity profile of the C protein generated using Parker Hydrophilicity tool available in IEDB resources is shown in Fig 3. The ‘y’ axis shows the amino acid residue of the capsid protein sequence and the score indicates the hydrophilicity. Two major regions with high hydrophilicity were visible, one at the amino (N)- terminal end spanning amino acid residues 1–45, and the other at the carboxy (C)- terminal end (70–100 aa). As regions with hydrophilic amino acids residues of a protein are generally surface exposed, such regions are potentially antigenic. These identified regions are agreeable to the regions on DENV2 capsid protein identified as hydrophilic, in a study done by AnandaRao (2005) [10] using Kyte and Dolittle hydrophilicity plot. They have identified 1–25 region of N terminus and 70–100 region of C terminus as hydrophilic. In both studies the central domain (40–70 aa) located between the above mentioned two hydrophilic regions, was discernible as a largely hydrophobic region. Hence this central region is less likely to be antigenic as compared to the two terminal regions.

The surface accesibilty of the C protein was predicted by the Emini Surface Accessibility tool available in IEDB resources (Fig 4). In agreement to the hydrophobicity nature of the capsid protein, again two main regions, in the N and C termini could be identified as surface accessible. Within these regions, patches of peptides covering, 3–8 a.a, 16–26 a.a, and 94–100 a.a are noticeably high with surface accessibility. The central hydrophobic region identified in the previous hydrophilicity profile is visible as a region with low surface accessibility in the current analysis as well.

As shown in Fig 5, Karplus & Schulz Flexibility profile visualizes highly flexible regions of capsid protein. The amino (N)-terminal end of the C protein seems to be more flexible especially the region spanning from ~3–42 a.a. Patches of highly flexible region could also be identified within the region between 70–100 a.a residues of the DENV capsid protein. The flexibility appears the lowest in the central region of the protein (between 40–70 a.a.)

Considering the results from all the three analysis, the N and C terminal regions of the C protein, the residues constituting approximately 1–40 a.a and 70–100 a.a could be considered rich with hydrophilicity, surface accessibility and flexibility. Such properties are characteristic of protein regions, which are antigenic. According to the analysis, the terminal regions of the capsid protein have the potential to produce antibodies if the C protein is encountered by B-cells. Further, these results were in line with the predicted conformational epitopes of the capsid, which have been predicted based on the tertiary structure of the protein. Seven conformational epitopes were predicted (Table 1), which are located within the same terminal regions identified as antigenic, according to the bioinformatics analysis described previously. It was also noted that the bulk of the residues in the predicted conformational epitopes are continuous. To further characterize the ability of these regions to induce antibody responses in the host, biochemical characterization of the antigenicity of the C protein was evaluated as described below.

**Table 1:** Predicted conformational epitopes of C protein.

Conformational epitopes (CEP)	#Residues	Number of residues	Score
CEP1	M1, N2, N3, Q4	4	0.943
CEP2	R98, R100, S101, V102, T103, M104, L105, L106, M107, L108, M109, P110, T111, A112, L113, A114	16	0.842
CEP3	R5, K7, T8, A9, R10, P11, S12, F13, N14, M15, L16, K17	12	0.780
CEP4	F33, S34, K35	3	0.614
CEP5	G36, L37, L38, S39, G40, Q41, G42, P43, M44	9	0.588
CEP6	S89, S90, N93, I94, M95, N96, R97	7	0.568
CEP7	K73, K74, N75	3	0.561

#Epitope residues are given with reference to DENV1 isolate AY713476.

Prediction tool: Ellipro, Cut-off score: 0.5.

### Indirect ELISA assays

To investigate the human antibody responses to C protein, sera collected from healthy volunteers previously infected with DENV were subjected to indirect ELISA assays. A peptide array representing the whole DENV2 C protein was tested for their binding to antibodies generated against the C protein.

The results are summarized in Table 2. A positive antibody response is an ELISA measurement that is above the cut-off value and, indicates the presence of antibodies against particular peptides. Three peptides {P1 (2–18 aa), P11 (79–95 aa) and P12 (86–101 aa)} showed high positive antibody responses to sera obtained from individuals exposed to only one of the 4 serotypes and people exposed to two serotypes. For P11 and P12, 100% of the sera from each serotype were positive, whereas for P1, 100% sera from DENV1, 3 & 4, and 83% sera from DENV2 were positive. Therefore, P1, P11 and P12 should constitute the highly antigenic conserved epitopes of the C protein. These peptides (P1, P11 and P12) are located on the N and C terminal regions (1–40 and 70–100 respectively) of the C protein, which are characterized to be highly hydrophilic, surface accessible and flexible regions according to our bioinformatics characterization and was predicted to include antigenic epitopes. Therefore, our bioinformatics prediction of potential antigenic regions of the C protein agrees well with the serology data.

**Table 2:** ELISA responses^*^ of capsid peptides.

	Sera Type	DENV2 Capsid Peptide ID (Position a.a.)													
		P1 (2–18)	P2 (8–25)	P3 (16–32)	P4 (23–40)	P5 (31–48)	P6 (39–56)	P7 (47–63)	P8 (55–73)	P9 (62–80)	P10 (71–89)	P11 (79–95)	P12 (86–101)	P13 (92–108)	P14 (99–114)
Sera infected with only one serotype	DENV1 serotype	**12** **100%**	0 0%	0 0%	0 0%	0 0%	0 0%	0 0%	0 0%	2 17%	1 8%	**12** **100%**	**12** **100%**	2 17%	0 0%
	DENV2 serotype	**10** **83%**	1 8%	1 8%	0 0%	0 0%	**12** **100%**	0 0%	0 0%	1 8%	1 8%	**12** **100%**	**12** **100%**	0 0%	2 17%
	DENV3 serotype	**12** **100%**	0 0%	0 0%	0 0%	0 0%	0 0%	0 0%	0 0%	0 0%	0 0%	**12** **100%**	**12** **100%**	0 0%	0 0%
	DENV4 Serotype	**12** **100%**	2 17%	0 0%	0 0%	0 0%	**6** **50%**	1 8%	0 0%	0 0%	**12** **100%**	**12** **100%**	**12** **100%**	0 0%	0 0%
Sera infected with two serotypes		**4** **33%**	0 0%	0 0%	0 0%	0 0%	**3** **25%**	0 0%	0 0%	1 8%	4 33%	**12** **100%**	**12** **100%**	0 0%	0 0%

*Number of sera samples giving positive responses out of 12 sera samples tested per serotype, and its percentage are shown.

Table 3 shows the mean OD values of the three peptides (P1, P11 and P12) which gave the highest positive response for all four serotypes in primary infections. No significant difference (P<0.05) was observed for the levels of antibody binding among the three peptides within a given serotype or across the serotypes.

**Table 3:** Mean OD_450nm_* values of positive peptides among four serotypes.

Positive peptides	Sera infected with only one serotype				Sera infected with two serotypes
	DENV1	DENV2	DENV3	DENV4	
P1	0.333 ± 0.06 (n = 12)	0.293±0.14 (n = 10)	0.318±0.05 (n = 12)	0.324±0.04 (n = 12)	0.316±0.039 (n = 4)
P11	0.314±0.06 (n = 12)	0.394±0.06 (n = 12)	0.304±0.05 (n = 12)	0.342±0.04 (n = 12)	0.351±0.040 (n = 12)
P12	0.321± 0.09 (n = 12)	0.424±0.03 (n = 12)	0.311±0.07 (n = 12)	0.324±0.04 (n = 12)	0.394±0.017 (n = 12)
P6	No positive response	0.245±0.03 (n = 12)	No positive response	0.153±0.00 (n = 6)	0.193±0.001 (n = 3)

*Mean OD_450nm_ values represent corrected values obtained after subtracting cut-off OD of each peptide.

(cut off values; P1 = 0.165, P6 = 0.147, P11 = 0.160, P12 = 0.156).

For the three peptides, P4 (23–40 aa), P5 (40–48 aa), P8 (54–71 aa), none of the sera samples showed any positive response. The latter two are located in the central hydrophobic, surface inaccessible less flexible region and therefore are not likely to be antigenic according to bioinformatic analysis.

The peptide, P10 (71–89 aa), which also falls well within the potential antigenic region according to the bioinformatics analysis, have not shown high positive antibody responses. Though it has been responsive for sera of individuals who responded to only one serotype, from three types (DENV1, DENV2 and DENV4), the percentage of responses is low (only 14% of sera) except in the case of DENV4 (100% of sera). With sera infected with two serotypes, its response is about 33%. For P6 (39–56 aa) on the other hand, though it falls in the central non-antigenic region, moderate levels of positive responses was observed with the sera of individuals who had been infected with DENV2 (100% sera), DENV4 (50% sera) and sera of individuals who responded to peptides of two serotypes (50% sera). This peptide therefore appears to be an exemption to the bioinformatics predictions of antigenicity. Moreover, unlike P1, P11 and P12, the antibody response for P6 appears specific for DENV2 as the epitope, which also belong to the homologous serotype, has been preferentially recognized by antibodies in sera infected with the homologous DENV2. For the remaining peptides, P2 (8–25 aa), P3 (16–32 aa), P7 (47–63 aa), P9 (62–79 aa), P13 (93–110 aa) and P14 (100–114 aa), the percentage of sera that showed a positive antibody responses was very low with both types of sera. These peptides are located either in the margins of identified potentially antigenic regions or in the non-antigenic central region.

When all the data from ELISA assays are analyzed together, the initially predicted potentially antigenic regions could be narrowed down. Out of the four peptides, P1 (2–18 a.a), P2 (8–25 a.a), P3 (16–32 a.a), and P4 (23–40 a.a), which partially or fully constitute the N terminal region (covering 1–40 a.a residue region) predicted to be antigenic by bioinformatics analysis, only P1 gave a high antibody response broadly across the serotypes while P4 failed to show any response. The partial overlapping of P2 and P3 with P1 may be the reason for the observed partial anybody responses for P2 and P3. Therefore, the most immunogenic region of the N terminus of the capsid protein seems to be within the amino acid resides from 2 to 18. The C terminal region of the protein, initially predicted to be antigenic is represented by the peptides P9 (62–80), P10 (71–89), P11 (79–95), P12 (86–101) and P13 (92–108). Out of these, only P11 and P12 have shown the high (100% of the sera samples) and broad (across all four serotypes) antibody responses. Therefore, the most immunogenic region of the DENV capsid C terminus seems to be between the amino acid residues 79 to 101, based on the ELISA results which were also supported by bioinformatics analysis. It should also note, however, since the capsid peptides subjected to the current ELISA assays are in the form of linear epitopes, interactions with the antibodies generated against the conformational epitopes, if any, may have not been fully formed. This could be one reason that some of the peptides, which were bioinformatically predicted to be antigenic, have failed to show positive antibody responses.

**Fig 6:**
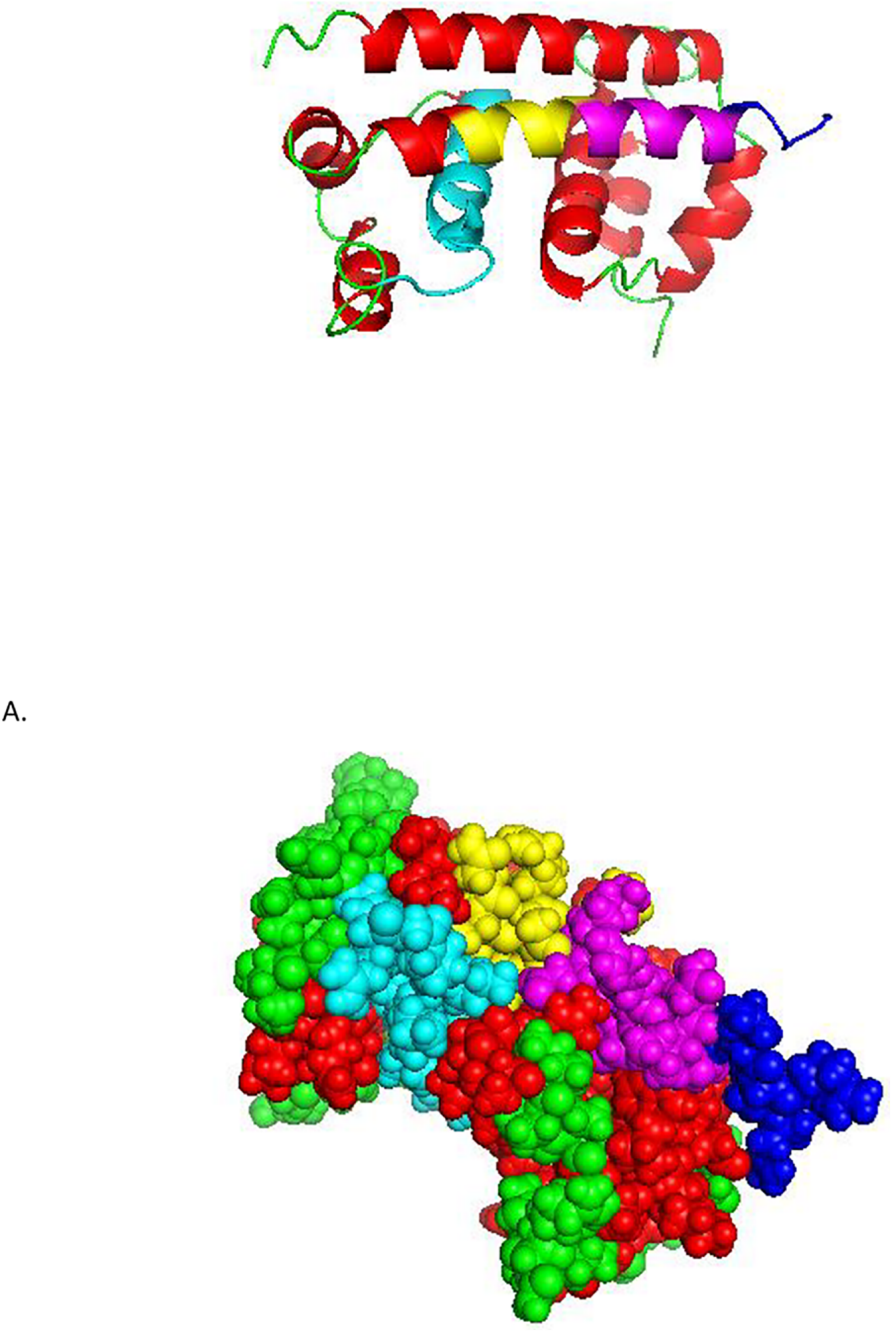
Map of epitopes P11, P12 and P6 on capsid dimer (P6: cyan, P11: yellow, P12: blue, overlapping region of P11 and P12: purple). A. Secondary structure of the capsid monomer. B. Spherical structure of the capsid monomer.*P1 sequence is not available in PDB 1R6R.

The above antigenic peptides of the capsid protein were then mapped to the solution structure of the protein (Fig 6). Peptides, P11 and P12, which constitute the antigenic region at the C terminus, as found out in the current study, falls in the alpha-helix 4, which is known as highly positively charged, solvent exposed, and has been suggested to interact with the viral genome [18]. P6 falls within the alpha-helix 2, which is largely hydrophobic and known to be involved in the dimerization of the capsid monomers. The P1 peptide constitutes the first 20 amino acids of C and this region appears to be flexible and was not included in the published structure of the dimer. Next, the C protein peptides were further analysed by using IEDB conservancy analysis (Table 4) to correlate the type specificity or the cross reactivity of the observed antibody responses with the sequence variability / similarity of the peptides. Out of those peptides that showed positive antibody responses, P11 and P12 showed a fairly high pan-serotype conservancy (≤59%), which is also reflected in their broad cross reactivity with the antibodies generated against all the four serotypes. The other broadly cross reactive immunogenic peptide, P1, did not comprise a high percentage of pan-serotype conservancy. This could imply that the residues in P1, which are conserved, are the most immunogenic of the peptide. The peptide P6 showed a moderate pan-serotype conservancy with high intra-serotype conservancies. This could explain the observed type specific antibody responses for P6.

**Table 4:** IEDB conservancy analysis of capsid peptides.

	Percentage conservancy (Min %)				
Protein/Peptide	Intra-serotype				Pan- serotype
	DENV1	DENV2	DENV3	DENV4	
Entire C protein					52%
P1: 2-NNQRKKARNTPFNMLKR-18	67%	72%	94%	78%	44%
P2: 8-RNTPFNMLKRERNRVSTV-25	72%	67%	100%	94%	44%
P3: 16-KRERNRVSTVQQLTKRF-32	94%	71%	94%	82%	59%
P4: 23-STVQQLTKRFSLGMLQGR-40	89%	72%	89%	72%	50%
P5: 31-RFSLGMLQGRGPLKLFMA-48	94%	78%	94%	83%	44%
P6: 39-GRGPLKLFMALVAFLRFL-56	94%	78%	100%	94%	56%
P7: 47-MALVAFRFLTIPPTAGI-63	89%	89%	94%	94%	72%
P8: 55-FLTIPPTAGILKRWGTIK-73	89%	89%	94%	100%	67%
P9: 62-GILKRWGTIKKSKAINVL-80	94%	89%	94%	94%	56%
P10: 71- IKKSKAINVLRGFRKEI-89	94%	94%	94%	94%	65%
P11: 79-NVLRGFRKEIGRMLNIL-95	88%	94%	94%	94%	65%
P12: 86-KEIGRMLNILNRRRRTA-101	71%	88%	94%	88%	59%
P13: 92-NILNRRRRTAGMIIMLI-108	65%	71%	88%	82%	36%
P14: 99-RTAGMIIMLIPTVMA-114	73%	67%	87%	87%	27%

## Discussion

The present study evaluated the antigenic potential of the C protein of DENV. Our results show strong agreement between C peptides predicted by different bioinformatic approaches to be antigenic and peptides recognized by antibodies in people infected with DENV. Although the capsid protein is not exposed on the viral surface, it stimulates a strong antibody response. The most antigenic peptides (P1, P11 and P12) were located at the flexible N-terminus (1–40 a.a) and the solvent exposed C-terminal region (70–100 a.a), of capsid protein. In detail, P1, P11 and P12 constitute amino acid residues 2 to 18, 79 to 95 and 86 to 101, respectively. Furthermore, the fact that antibodies bound to peptides that were computationally predicted to be antigenic and surface exposed on the intact protein suggests that the intact native protein is activating B-cells.

Our results agree well with C protein peptides found to be immunogenic in other studies. The N terminal P1 peptide (2–18 a.a) overlaps with the N-terminal capsid (DENV2) epitope, 9-RNTPFNMLKRE-19, reported by Bulich and Aaskov [19]. Puttikhunt et al. [8] produced monoclonal and polyclonal antibodies against the capsid protein, and observed that antibodies specifically recognized the first 20 amino acids of the DENV2 C protein. Another study done by AnandaRao et al. [10] identifies three peptides (2-NNQRKKARN-10, 82-LRGFR-85 and 91-MLNILNRRR-99) on DENV2 C protein that were recognized by human antibodies and all these three peptides overlap with the main peptides, P1 (2–18 a.a), P11 (79–95 a.a) and P12 (86–101 a.a) identified in the current study.

A major strength of our study is that we evaluated the C protein antibody response using immunesera from people with well-defined infections with different DENV serotypes. The above antigenic N and C terminal peptides of DENV2 C protein were efficiently recognized by immunesera from people exposed to different DENV serotypes demonstrating that the epitopes are conserved between serotypes and targets of DENV serotype cross-reactive antibodies. In addition, as reported by AnandaRao et al. these peptides show considerable divergence from the corresponding regions of two other common flaviviruses (Japanese Encephalitis Virus (JEV) and Yellow Fever Virus (YFV), supporting the use of these peptides for serodiagnosis of DENV infections [10].

In contrast to the P1, P11 and P12 peptides, the P6 peptide showed positive antibody responses preferentially to sera of individuals who had memory T cell responses to only DENV2 serotype. The results suggest that the capsid epitope represented by the P6 peptide, being a DENV2 specific one. It would be worth looking in to the antibody responses of the corresponding peptide sequences from DENV 1, 3 and 4, if they are also specific to sera of individuals who had memory T cell responses to the homologous serotype. Such an epitope could be a potential target as a type detection marker in dengue diagnosis.

The usability of the above peptides from dengue capsid protein, as dengue group or serotype detection markers, is a question to be evaluated further, with more ELISA assays using sera derived from other flaviviral infections. Whether those epitopes induce antibodies that cloud be involved in protective or pathogenic responses by mechanism such as Antibody-Dependent Cellular Cytotoxicity (ADCC), the formation of immune complexes or complement activation is also worth considering. Such a scenario would shed light in to the significance of dengue capsid protein in disease progression.

## Conclusions

In this study, using the DENV2 C protein as a model, we demonstrate strong concordance between peptides predicted by bioinformatics to be antigenic and individual peptides recognized by human dengue immunesera. The DENV C protein stimulates robust antibody responses even though the protein is not exposed on the viral surface. The N and C terminal regions of C protein contains antibody epitopes that are conserved between serotypes and efficiently recognized by people exposed to primary and secondary infections with different serotypes. Unlike the N and C-terminal regions, the central region of C protein has a peptide epitope that was mainly targeted by serotype-specific antibodies. Our results offer new insight into the human antibody response to an internal DENV structural protein.

## Supporting information

S1 Table Demographic information of healthy volunteers infected with only one DENV serotype. (DOCX)

S2 Table Demographic information of healthy volunteers infected with two DENV serotypes. (DOCX)
