# Supplementary material for: Natural antibody responses to the capsid protein in sera of Dengue infected patients from Sri Lanka
Source: PLoS One. 2017 Jun 5;12(6):e0178009. doi: 10.1371/journal.pone.0178009 (PMC5459338; doi:10.1371/journal.pone.0178009)
Supplement: S1 Table — (DOCX) [file pone.0178009.s001.docx]

**S1 Table: Demographic information of healthy volunteers infected with only one DENV serotype**

| **DENV serotype infected with (number of samples)** | **Age group**  **(number of participants)** | **Gender** | **Asymptomatic(Asy)/**  **Symptomatic (Sym)** | **Exposure to JE** |
| --- | --- | --- | --- | --- |
| DENV1  (n=12) | 10-20=0  20-30=1  30-40=4  40-50=3  50-60=3  60-70=1 | 6-Males  6-Females | Asy -10  Sym-1 | All participants have not been exposed to JE vaccine |
| DENV2  (n=12) | 10-20=0  20-30=3  30-40=7  40-50=0  50-60=0  60-70=2 | 7-Males  5-Females | Asy -11  Sym-2 | All participants have not been exposed to JE vaccine |
| DENV3  (n=12) | 10-20=2  20-30=3  30-40=3  40-50=2  50-60=2  60-70=0 | 8-Males  4-Females | Asy -12  Sym-0 | Two participants have been exposed to JE vaccine (age 10, 12 ). Others have not been exposed to the vaccine. |
| DENV4  (n=12) | 10-20=1  20-30= 6  30-40=1  40-50=0  50-60=0  60-70=4 | 9-Males  3-Females | Asy -12  Sym-0 | One participant has been exposed to JE vaccine (age10 ). Others have not been exposed to the vaccine |
