# Supplementary material for: Natural antibody responses to the capsid protein in sera of Dengue infected patients from Sri Lanka
Source: PLoS One. 2017 Jun 5;12(6):e0178009. doi: 10.1371/journal.pone.0178009 (PMC5459338; doi:10.1371/journal.pone.0178009)
Supplement: S2 Table — (DOCX) [file pone.0178009.s002.docx]

| **DENV serotype infected with (number of samples)** | **Age group**  **(number of participants)** | **Gender** | **Asymptomatic(Asy)/**  **Symptomatic (Sym)** | **Exposure to JE** |
| --- | --- | --- | --- | --- |
| DENV1 and DENV4  (n=2) | 10-20=0  20-30=0  30-40=2  40-50=0  50-60=0  60-70=0 | 0-Males  2-Females | Asy -2  Sym-0 | All participants have not been exposed to JE vaccine |
| DENV1 and DENV3  (n=2) | 10-20=0  20-30=1  30-40=0  40-50=1  50-60=0  60-70=0 | 0-Males  2-Females | Asy -1  Sym-1 | All participants have not been exposed to JE vaccine |
| DENV2 and DENV3  (n=1) | 10-20=0  20-30=0  30-40=1  40-50=0  50-60=0  60-70=0 | 1-Males  0-Females | Asy -0  Sym-1 | All participants have not been exposed to JE vaccine |
| DENV2 and DENV4  (n=4) | 10-20=0  20-30= 0  30-40=3  40-50=1  50-60=0  60-70=0 | 4-Males  0-Females | Asy -2  Sym-2 | One participant has been exposed to JE vaccine (age 31). Others have not been exposed to the vaccine |
| DENV3 and DENV1  (n=2) | 10-20=0  20-30= 0  30-40=0  40-50=1  50-60=1  60-70=0 | 1-Males  1-Females | Asy -0  Sym-2 | All participants have not been exposed to JE vaccine |
| DENV3 and DENV4  (n=1) | 10-20=0  20-30= 0  30-40=0  40-50=1  50-60=0  60-70=0 | 0-Males  1-Females | Asy -0  Sym-1 | All participants have not been exposed to JE vaccine |

**S2 Table: Demographic information of healthy volunteers infected with two DENV serotyoes**
